# Usefulness of Blood Rheology as a Predictor of Primary Cardiovascular Disease Events in Patients With Stage G3 Chronic Kidney Disease

**DOI:** 10.14740/jocmr6541

**Published:** 2026-04-15

**Authors:** Takashi Hitsumoto

**Affiliations:** Hitsumoto Medical Clinic, 2-7-7, Takezakicyou, Shimonoseki City, Yamaguchi 750-0025, Japan. Email: thitsu@jcom.home.ne.jp

**Keywords:** Blood rheology, Whole blood passage time, Chronic kidney disease stage G3, Cardiovascular events, Augmentation index, Skin autofluorescence, Renin–angiotensin system inhibitor, Prospective study

## Abstract

**Background:**

Several studies have highlighted that impairment of blood rheology plays a crucial role in the development of cardiovascular disease (CVD) and arteriosclerosis. However, the value of blood rheology as a potential predictor of the development of CVD in patients with chronic kidney disease (CKD) remains unknown. The objective of this prospective study was to investigate the utility of blood rheology using whole blood passage time (WBPT) as a predictor of primary CVD events in patients with stage G3 CKD, which is often encountered in daily practice.

**Methods:**

This study involved 417 outpatients with stage G3 CKD (male/female: 144/273) without history of CVD. WBPT was measured by microchannel array flow analyzer as a commercial device. Subsequently, the author evaluated the effectiveness of WBPT for the prediction of primary CVD events, which were defined as major adverse cardiovascular events (MACEs, i.e., cardiovascular death, nonfatal ischemic heart disease, and non-fatal ischemic stroke).

**Results:**

Patients were assigned into two groups according to the WBPT cut-off value, which was estimated by receiver operating characteristic curve analysis: low (group L, WBPT ≤ 74.3 s; n = 255) or high (group H, WBPT > 74.3 s; n = 162). During the median follow-up period of 76 months (range: 2–96 months), MACEs occurred in 74 patients (group L: n = 17 (6.7%) vs. group H: n = 57 (35.2%); P < 0.001, log-rank test). Multivariate Cox regression analysis revealed that patients in group H were at a significantly higher risk of developing MACEs than those in group L (hazard ratio: 3.89; 95% confidence interval: 2.12–7.10; P < 0.001).

**Conclusions:**

The present findings showed that impairment of blood rheology, determined using WBPT, is predictive of primary CVD events in patients with stage G3 CKD. Further large-scale studies are warranted to confirm whether various interventions can reduce the incidence of primary CVD events with improved WBPT in patients with stage G3 CKD.

## Introduction

Several studies have highlighted that impairment of blood rheology plays an important role in the development of cardiovascular disease (CVD) and arteriosclerosis [1, 2]. Numerous methods are used for the evaluation of blood rheology; among those, a commercially available device using microscope images, termed microchannel array flow analyzer (MC-FAN), has been utilized for the measurement of whole blood passage time (WBPT) [3]. The MC-FAN assesses blood rheology through the flow of the patient’s whole blood into an artificial vascular model. It has been shown that this method is simple and reliable; moreover, clinical evidence has demonstrated the significance of WBPT as an indicator of the risk of developing CVD [4–8].

In recent years, the incidence of chronic kidney disease (CKD) has been increasing worldwide due to the aging population and increase in lifestyle-related diseases (e.g., diabetes mellitus, dyslipidemia, and hypertension) [9, 10]. Additionally, several studies reported that CKD is closely associated with the incidence of CVD [11, 12]. An estimated glomerular filtration rate (eGFR) < 60 mL/min/1.73 m^2^ is indicative of stages G3–5 CKD. In clinical practice, the number of patients with stage G3 CKD is markedly higher than that of patients with stage G4 or G5 disease [13, 14]. Conversely, previous studies have reported that the incidence of CVD events in CKD in the G4–5 period is high [15]. Therefore, it would be clinically meaningful if we could detect high-risk cases of CVD corresponding to stage G4–5 CKD in patients with stage G3 CKD, which are often encountered in daily practice. The aim of this prospective study was to investigate the clinical utility of blood rheology using WBPT as a predictor of primary CVD events in patients with stage G3 CKD.

## Materials and Methods

### Patients

This study is a prospective study at a single center unit. WBPT measurement was blinded and also performed standardized across patients. From July 2017 to June 2019, 450 consecutive stage G3 CKD patients without history of CVD visited the Hitsumoto Medical Clinic, Yamaguchi Prefecture, Japan. Of those, 29 patients who lacked baseline clinical data including WBPT and four patients who failed to provide consent for their participation in this study were excluded. Finally, 417 outpatients (144 males (34.5%) and 273 females (65.5%)) were prospectively enrolled. The eGFR was measured using a formula developed for the Japanese population [16]. For patient classification, an eGFR ranging 30–59 mL/min/1.73 m^2^ denoted stage G3 CKD.

### Ethical considerations

The study protocol was approved by the Institutional Review Board of Hitsumoto Medical Clinic (date of approval: June 14, 2017; Approval Number: HMC-2017-7). The clinical study was conducted in compliance with the ethical principles of the Declaration of Helsinki, which is the ethical principle of medical research involving human beings.

### Evaluation of blood rheology

Blood rheology was assessed by measuring the WBPT using a commercialized MC-FAN HR300 rheometer (MC Healthcare Inc., Tokyo, Japan), as previously described [3, 7]. WBPT measurements were performed on the morning of the same day as blood collection by healthcare professionals who were not informed of the study’s content beforehand. The microchannel transit time of saline (100 µL) was initially measured as a control. Subsequently, similar measurements were performed on whole blood heparinized samples (100 µL) obtained from patients. The WBPT measurements were normalized to those of the saline transit time and were measured within 60 min following sample collection. The configuration of the microchannels was as follows: length, 30 µm; width, 7 µm; and depth, 4.5 µm. The reliability of the present WBPT measurements was better than previously reported (i.e., inter- and intra-assay coefficients of variation: 8% and 5%, respectively) [5].

### Evaluation of clinical parameters

The degree of obesity was assessed by body mass index, and patients with 25.0 kg/m^2^ or more were considered obesity [17]. Other CVD risk factors such as smoking, hypertension, dyslipidemia, and diabetes mellitus have been decided using previous report [8]. Blood or urine collection was collected after fasting for more than 12 h. Hematocrit, serum creatinine levels, high-sensitivity C-reactive protein levels, serum lipid levels, blood glucose levels, and urinary albumin levels were measured using standard methods. Albuminuria was defined as ≥ 30 mg/g Cr of urinary albumin levels. Skin autofluorescence (AF) is known to be a marker of advanced glycation end products (AGEs) *in vivo*; in this analysis, it was measured on the medial forearm of the patients using commercially available devices (AGE Reader; DiagnOptics, Groningen, Netherlands) in a manner similar to previous reports [18, 19]. The radial augmentation index (r-AIx) (marker of arterial reflected waves) was calculated using a tonometry-based device (HEM-9010AI, OMRON Healthcare Co., Ltd., Kyoto, Japan), as in other studies [20, 21]. Oral medications, such as renin–angiotensin system (RAS) inhibitor statin, and anti-diabetes medications were also investigated.

### Statistical analysis

The commercialized software Stat View-J 5.0 (HULINKS Inc., Tokyo, Japan) and MedCalc (MedCalc Software, Ostend, Belgium) were used for the statistical analysis. Receiver operating characteristic curves were created, and the maximum Youden index was determined [22]. Continuous variables were expressed as means and standard deviations or median (interquartile range). Student’s *t*-test or Mann–Whitney U test was used to perform comparisons between groups. Kaplan–Meier analysis was conducted to produce event-free survival curves; differences between these curves were assessed using a log-rank test. Multivariate analysis was performed using multivariate Cox regression analysis. First, in selecting explanatory variables for the multivariate Cox regression analysis, the number of major adverse cardiovascular events (MACEs) in this study (n = 74) was taken into consideration, and statistical analysis was performed to select seven or eight covariates. Specifically, 11 factors that showed differences between group H and group L, and/or depending on the presence or absence of MACEs, were adjusted each other using stepwise analysis, and eight factors (i.e., age, WBPT, diabetes, eGFR, cutaneous AF, r-AIx, albuminuria, and use of RAS inhibitors) were selected as covariates for the multivariate Cox regression analysis in this study. Cut-off values for MACEs of age, skin AF, and r-AIx are decided by receiver operating characteristic curve analysis. P values < 0.05 denoted statistically significant differences.

## Results

### Follow-up and grouping

**Figure 1:**
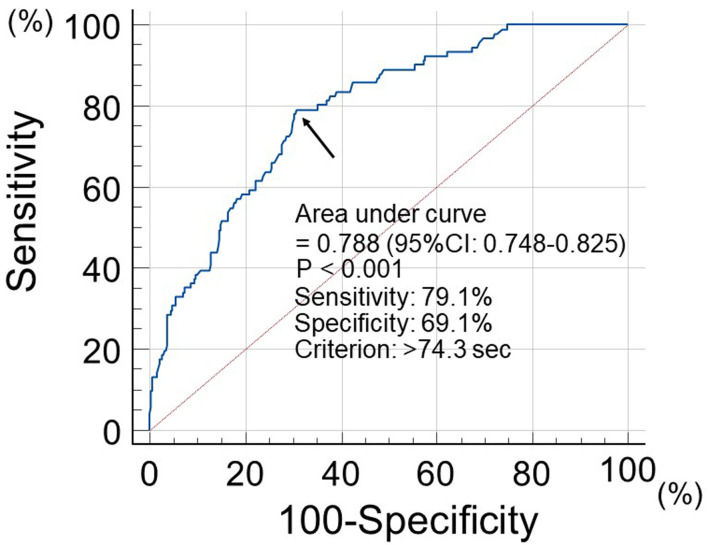
Prediction value of MACEs incidence at follow-up period using WBPT. Receiver operating characteristic curve analysis indicated that a cut-off value for WBPT of 74.3 s yielded the largest area under the curve to predict MACEs. Arrows indicate the optimal cut-off point. MACEs: major adverse cardiovascular events; WBPT: whole blood passage time.

The patients were followed up until June 2025. The endpoint of this study was the occurrence of primary CVD events, which were defined as MACEs. These MACEs were a composite of cardiovascular death, nonfatal ischemic heart disease, and non-fatal ischemic stroke. Patients were assigned into two groups according to the cut-off value of the WBPT, which was estimated by receiver operating characteristic curve analysis for MACEs (Fig. 1): low (group L, WBPT ≤ 74.3 s; n = 255) or high (group H, WBPT > 74.3 s; n = 162). The author’s previous report in patients with traditional cardiovascular risk factors also showed WBPT values for MACEs similar to those in this study [8].

### Clinical characteristics

Table 1 shows the clinical characteristics of the studied groups. The mean WBPT for groups L and H was 55.9 and 88.5 s, respectively. Group H was associated with a significantly higher frequency of smoking, incidence of diabetes mellitus, hematocrit, serum triglyceride levels, skin AF, incidence of albuminuria, and r-AIx compared with group L. However, group H had significantly lower eGFR, high-density cholesterol levels, and frequency of RAS inhibitor use than group L.

**Table 1:** Clinical Characteristics of the Studied Groups

Characteristics	Overall	Group L	Group H	P value
n (male/female)	417 (144/273)	255 (91/164)	162 (53/109)	0.535
Age (years)	72 ± 12	71 ± 10	75 ± 13	0.062
WBPT (s)	68.6 ± 11.1	55.9 ± 11.0	88.5 ± 11.2	< 0.001
Risk factors				
Obesity, n (%)	124 (30)	76 (30)	48 (30)	0.964
Current smoker, n (%)	74 (18)	36 (14)	38 (24)	0.014
Hypertension, n (%)	298 (72)	181 (71)	117 (72)	0.785
Dyslipidemia, n (%)	293 (70)	178 (70)	115 (71)	0.797
Diabetes mellitus, n (%)	134 (32)	67 (26)	67 (41)	0.001
Clinical parameters				
eGFR (mL/min/1.73 m^2^)	47 ± 6	50 ± 6	44 ± 5	< 0.001
LDL cholesterol (mg/dL)	129 ± 35	128 ± 35	131 ± 35	0.414
Triglyceride (mg/dL)	122 ± 66	108 ± 61	143 ± 67	< 0.001
HDL cholesterol (mg/dL)	62 ± 15	64 ± 14	59 ± 14	0.001
FBG (mg/dL)	116 ± 27	114 ± 27	119 ± 27	0.125
hs-CRP (mg/L)	0.80 (0.30–1.50)	0.70 (0.30–1.20)	0.86 (0.29–1.97)	0.055
Skin AF (AU)	2.7 ± 0.5	2.6 ± 0.5	2.8 ± 0.5	< 0.001
Albuminuria, n (%)	213 (51)	111 (44)	102 (63)	< 0.001
r-AIx (%)	87 ± 11	85 ± 9	89 ± 12	< 0.001
Medication				
RAS inhibitor, n (%)	156 (37)	106 (42)	50 (31)	0.027
Statin, n (%)	148 (36)	96 (38)	52 (32)	0.249
Sulfonylurea, n (%)	77 (19)	45 (18)	32 (20)	0.591
Metformin, n (%)	62 (15)	35 (14)	27 (17)	0.412
DPP-4 inhibitor, n (%)	75 (18)	41 (16)	34 (21)	0.204

Continuous variables were expressed as means and standard deviations or median (interquartile range). AF: autofluorescence; AU: arbitrary unit; DPP-4: dipeptidyl peptidase-4; eGFR: estimated glomerular filtration rate; FBG: fasting blood glucose; HDL: high-density lipoprotein; hs-CRP: high-sensitivity C-reactive protein; LDL: low-density lipoprotein; r-AIx: radial augmentation index; RAS: renin–angiotensin system; WBPT: whole blood passage time.

### Kaplan–Meier curve analysis

**Figure 2:**
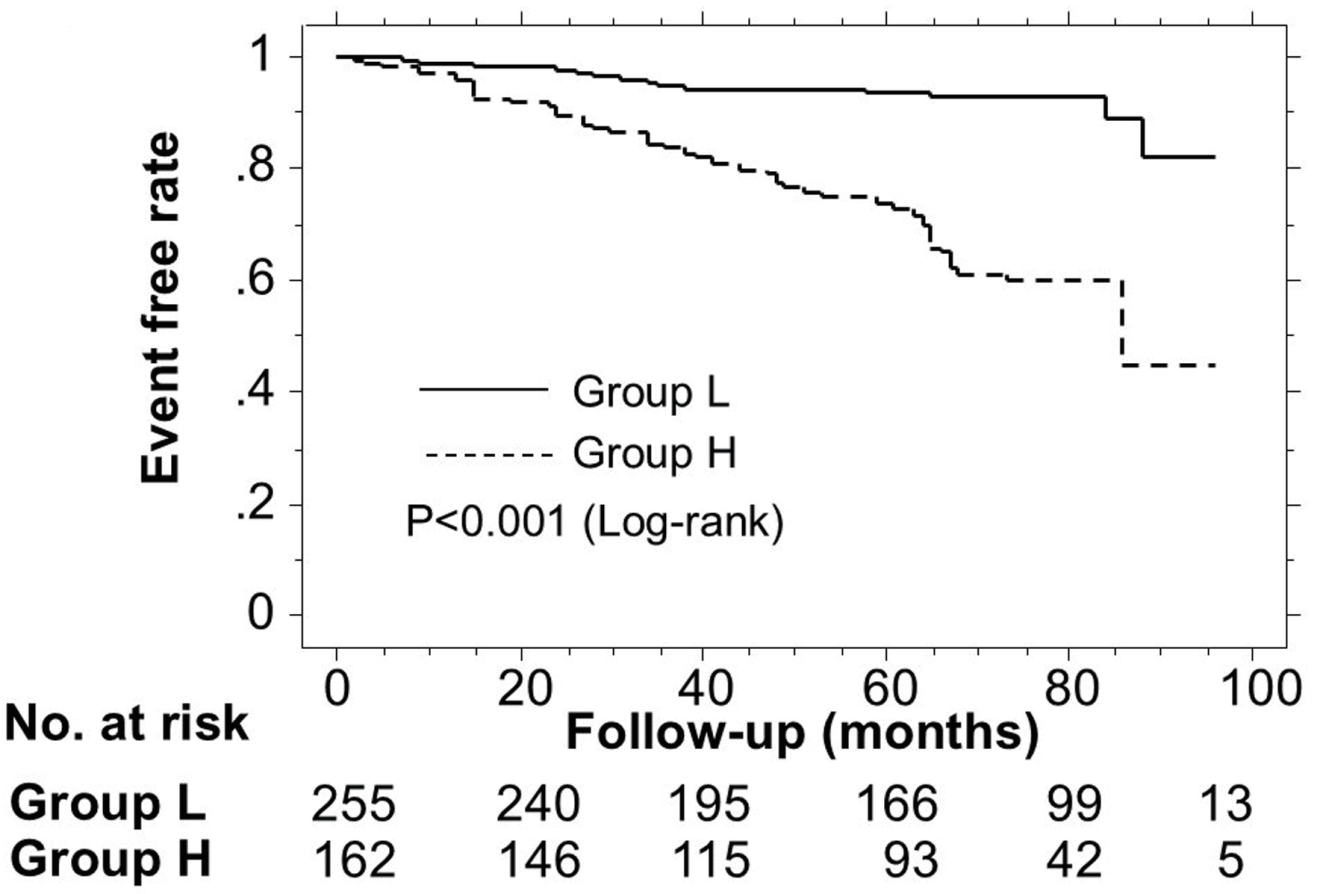
Kaplan–Meier curve analysis for the incidence of MACEs. The Kaplan–Meier curve confirmed that group H had a significantly higher incidence of MACEs compared with group L (log-rank test, P < 0.001). MACEs: major adverse cardiovascular events.

Figure 2 shows the Kaplan–Meier curve for the incidence of MACEs. The median follow-up was 76 months (range: 2–96 months). During the follow-up, 74 patients developed MACEs (group L: 17 cases (6.7%)); group H: 57 cases (35.2%)). Group H had a significantly higher incidence of MACEs versus group L (log-rank test, P < 0.001).

### Relationship between MACEs and clinical parameters

Table 2 presents the association between the presence or absence of MACEs and each clinical parameter. Age, incidence of diabetes mellitus, fasting blood glucose levels, skin AF, frequency of albuminuria, and r-AIx were significantly higher in patients who experienced MACEs compared with those who did not. However, patients who experienced MACEs had significantly lower eGFR and RAS inhibitor use than those who did not.

**Table 2:** Parameters at Registration of Patients With and Without MACEs

Characteristics	MACEs (−)	MACEs (+)	P value
n (male/female)	343 (119/224)	74 (25/49)	0.798
Age (years)	71 ± 12	77 ± 11	< 0.001
Obesity, n (%)	104 (30)	20 (27)	0.575
Current smoker, n (%)	57 (17)	17 (22)	0.051
Hypertension, n (%)	240 (70)	58 (78)	0.147
Dyslipidemia, n (%)	242 (71)	51 (69)	0.780
Diabetes mellitus, n (%)	89 (26)	45 (61)	< 0.001
eGFR (mL/min/1.73 m^2^)	48 ± 6	44 ± 7	< 0.001
LDL cholesterol (mg/dL)	129 ± 34	130 ± 35	0.691
Triglyceride (mg/dL)	119 ± 66	139 ± 62	0.051
HDL cholesterol (mg/dL)	63 ± 15	59 ± 14	0.072
FBG (mg/dL)	114 ± 27	116 ± 27	< 0.001
hs-CRP (mg/L)	0.80 (0.30–1.50)	0.80 (0.27–1.60)	0.624
Skin AF (AU)	2.6 ± 0.5	3.0 ± 0.5	< 0.001
Albuminuria n (%)	162 (47)	51 (69)	< 0.001
r-AIx	86 ± 10	93 ± 11	< 0.001
RAS inhibitor, n (%)	140 (41)	21 (21)	0.002
Statin, n (%)	127 (37)	21 (28)	0.159
Sulfonylurea, n (%)	63 (18)	14 (19)	0.912
Metformin, n (%)	50 (15)	12 (16)	0.720
DPP-4 inhibitor, n (%)	58 (17)	17 (23)	0.219

Continuous values are mean ± SD. AF: autofluorescence; AU: arbitrary unit; DPP-4: dipeptidyl peptidase-4; eGFR: estimated glomerular filtration rate; FBG: fasting blood glucose; HDL: high-density lipoprotein; hs-CRP: high-sensitivity C-reactive protein; LDL: low-density lipoprotein; MACEs: major adverse cardiovascular events; r-AIx: radial augmentation index; RAS: renin–angiotensin system.

### Multivariate Cox regression analysis

Table 3 shows the results of the multivariate Cox regression analysis for MACEs. Among the eight factors, six factors (i.e., WBPT, diabetes mellitus, age, RAS inhibitor use, skin AF, and r-AIx) exhibited a significant hazard ratio for MACEs. However, the multivariate Cox regression analysis did not identify eGFR and albuminuria as significant factors for MACEs.

**Table 3:** Multivariate Cox Regression Analysis for MACEs

Variables	HR	95% CI	P value
Group H (vs. group L)	3.89	2.12–7.10	< 0.001
Diabetes mellitus	2.11	1.26–4.54	0.004
Age (≥ 75 years)	1.98	1.35–3.91	0.006
RAS inhibitor	0.51	0.27–0.88	0.018
Skin AF (≥ 2.7 AU)	1.95	1.12–3.40	0.019
r-AIx (≥ 88%)	1.78	1.03–3.10	0.041
Albuminuria	1.22	0.51–1.86	0.353
eGFR (stage 3A vs. stage 3B)	1.12	0.67–2.21	0.536

AF: autofluorescence; AU: arbitrary unit; CI: confidence interval; eGFR: estimated glomerular filtration rate; HR: hazard ratio; MACEs: major adverse cardiovascular events; r-AIx: radial augmentation index; RAS: renin–angiotensin system.

## Discussion

This study examined the clinical utility of blood rheology as a predictive indicator of CVD events in stage G3 CKD patients who are frequently detected in daily practice. The results of this analysis highlighted WBPT, an indicator of blood rheology, as an independent predictor of MACEs development in such patients. Therefore, it is hypothesized that patients who are at high risk of experiencing CVD events groups can be identified by measuring the WBPT as a marker of blood rheology. Similar to this study, previous studies have demonstrated the predictive value of classical CVD risk factors (aging and diabetes mellitus) for the occurrence of CVD events in CKD patients [23–25]. In addition, relatively novel CVD risk factors (skin AF and r-AIx) were also selected as independent predictors of MACEs development in this study. Furthermore, skin AF and r-AIx were significantly related to WBPT in univariate analysis. However, RAS inhibitor use was selected as a predictor to inhibit the onset of MACEs.

### Significance of WBPT as a CVD risk factor in CKD

The WBPT method uses the whole blood of the patient to determine blood rheology in the artificial blood vessel. Factors of whole blood rheology include leukocyte adhesion, erythrocyte deformation, platelet aggregation, and plasma viscosity; it is speculated that WBPT is defined by these factors. However, several studies have reported that the abovementioned individual factors play a role in the onset of CVD events [26–28]. Therefore, previous studies suggest that WBPT can be a predictor of MACEs in stage G3 CKD patients. Researchers have emphasized that low eGFR and albuminuria are significant factors in the development of CVD events [29, 30]. This study revealed that these factors differed significantly between patients with and without MACEs; however, this statistically significant difference was not observed in the multivariate Cox regression analysis. The importance of low eGFR and albuminuria in the development of CVD events is obvious. In addition, the number of cases in this study was relatively small, and as a result, it is presumed that no significant differences in eGFR and albuminuria were observed. However, a statistically significant association was found between high WBPT and low eGFR and albuminuria in univariate analysis, although it is not clear as to the mechanism. Thus, the results of this study are considered that the increase in WBPT on the background of the low eGFR and the albuminuria is a more important factor for the development of MACEs in stage G3 CKD patients.

### Relationships between AGEs and blood rheology

Meerwaldt et al performed skin biopsy samples collected from the same site and revealed a significant correlation between skin AF values and the levels of AGEs [31]. Therefore, skin AF is measured at a site where capillaries are located. However, the artificial blood vessel used in MC-FAN also assumes the presence of capillaries. Hence, it is presumed that skin AF represents the accumulation of AGEs in the blood vessel wall caused by the impairment of blood rheology in the capillaries. Moreover, several researchers have reported that AGEs influence blood rheology through mechanisms of abnormal blood cell function [32–34]. These results indicated an association between AGEs and blood rheology. However, some studies have shown the relationship between skin AF and the AGEs in heart and brain. Hofmann et al indicated that skin AF was significantly associated with AGEs-modified cardiac tissue collagen [35]. Also, Ohnuki et al clarified that skin AF was significantly linked to chronic cerebral infarction, including lacunar infarction, which is caused by the occlusion of a small-sized vessel [36]. Furthermore, several investigators have reported the importance of the vasa vasorum flow in arterial function or plaque vulnerability [37–39]. Hemorheological impairment in the vasa vasorum, which reflects high WBPT, may increase the risk of arterial dysfunction or plaque rupture in the heart and brain. Therefore, the significant association between skin AF and WBPT detected in this study suggested a close relationship between AGEs and impairment of blood rheology in important organs related to CVD events (heart and brain) in patients with stage G3 CKD.

### Relationships between arterial reflected waves and blood rheology

Some studies on CKD have shown that AIx is associated with the presence of CVD [40, 41]. Additionally, the present investigation revealed for the first time that r-AIx is an independent predictor of MACEs in patients with stage G3 CKD. However, in the present study, the r-AIx was significantly higher in group H versus group L. Therefore, impairment of blood rheology may have caused an increase in arterial reflected waves. Previous reports have proposed microvascular dysfunction as a factor influencing the increase in arterial reflection waves [42, 43]. Nonetheless, the author has previously reported a significant association between WBPT and the renal resistive index [44]. The renal resistive index is an indicator of microvascular resistance in the kidney. Thus, an increase in renovascular resistance due to impairment of blood rheology in renal small-sized vessels may lead to an increase in arterial reflection waves; consequently, CVD events occurred.

### Medications and blood rheology

Many studies indicated that RAS inhibitor use is effective in reducing the progression of CKD [45, 46]. Additionally, researchers have demonstrated that RAS inhibitor use reduces the development of CVD events in patients with CKD [47, 48]. Moreover, findings of this study indicated that RAS inhibitor use was independently associated with a reduction in the incidence of MACEs in patients with stage G3 CKD. Moreover, studies demonstrated that RAS inhibition is effective in improving the factors of blood rheology [49, 50]. However, in the present study, group L used 42% of RAS inhibitor, while group H used 31% of them (P = 0.027). Thus, the use of RAS inhibitor in patients with high WBPT and improved blood rheology may exert a further inhibitory effect on primary CVD events. Thus, intervention trials using RAS inhibitor that focus on blood rheology in stage G3 CKD patients are warranted. In recent years, the efficacy of sodium glucose cotransporter 2 (SGLT2) inhibitors in preventing the development of CVD events in patients with CKD has been demonstrated [51, 52]. However, several studies have shown that SGLT2 inhibitor has a beneficial effect on blood rheology [53, 54]. Although none of the patients used SGLT2 inhibitor at baseline, further research on the benefits of SGLT2 inhibitor in preventing the CVD events in stage G3 CKD patients is desired from the perspective of WBPT. Existing reports have shown a significant relationship between WBPT and smoking [55, 56]. In addition, Shimada et al reported that WBPT significantly decreased with smoking cessation [56]. However, in this study, no significant association was found between smoking habits and the incidence of primary CVD events, although group H had a significantly higher number of smokers compared to group L. Therefore, the results of this study suggest that smoking may contribute to the onset of CVD events in stage G3 CKD patients through a decrease in blood rheology. In recent years, the efficacy of varenicline in smoking cessation treatment has been documented [57, 58]. Therefore, for smokers with high WBPT, proactive interventional treatment including varenicline is expected to help prevent CVD through improved blood rheology in these patients.

### Limitations

The limitations of this study should be acknowledged. Firstly, this analysis was performed at a single center in Japan with relatively small number. Consequently, the findings may not be generalizable to other populations. Secondly, exclusion of patients without baseline clinical data including WBPT may introduce selection bias. Thirdly, in this study, the Japanese-specific eGFR equation was used, so its application to other ethnic groups is limited, and this should be recognized. In addition, the fact that no significant differences were observed in eGFR and albuminuria in the multivariate Cox regression analysis contradicts existing literature, and this point requires careful interpretation. Finally, while WBPT appears promising, the conclusions may overstate its utility as a predictor without external validation or intervention data. In addition, WBPT was measured only once at baseline, which limits conclusions about causality and longitudinal changes. Therefore, large, multicenter prospective trials are warranted to determine whether variations in the WBPT contribute to the development of primary CVD events in patients with stage G3 CKD, including interventional trials such as RAS inhibitor use, SGLT2 inhibitor use, and smoking cessation.

### Conclusions

According to the present findings, impairment of blood rheology, determined using WBPT, is predictive of primary CVD events in patients with stage G3 CKD. Further large-scale studies are warranted to confirm whether various interventions can reduce the incidence of primary CVD events with improved WBPT in patients with stage G3 CKD.

## Data Availability

The author declares that data supporting the findings of this study are available within the article.

## Abbreviations

AF: autofluorescence
AGEs: advanced glycation end products
CKD: chronic kidney disease
CVD: cardiovascular disease
eGFR: estimated glomerular filtration rate
MACEs: major adverse cardiovascular events
MC-FAN: microchannel array flow analyzer
r-AIx: radial augmentation index
RAS: renin–angiotensin system
SGLT2: sodium glucose cotransporter 2
WBPT: whole blood passage time
